# Supplementary material for: Observation of a thermoelectric Hall plateau in the extreme quantum limit
Source: Nat Commun. 2020 Feb 25;11:1046. doi: 10.1038/s41467-020-14819-7 (PMC7042294; doi:10.1038/s41467-020-14819-7)
Supplement: Supplementary file 1 — Supplementary Information [file 41467_2020_14819_MOESM1_ESM.pdf]

Supplementary Information for "Observation of a thermoelectric Hall plateau in the extreme quantum limit"

Wenjie Zhang,[1,*] Peipei Wang,[2,*] Brian Skinner,[3,4,*] Ran Bi,[1] Vladyslav Kozii,[3,5,6] Chang-Woo Cho,[2] Ruidan Zhong,[7] John Schneeloch,[7] Dapeng Yu,[2] Genda Gu,[7] Liang Fu,[3,†] Xiaosong Wu,[1,8,†] and Liyuan Zhang[2,†]

[1]*State Key Laboratory for Artificial Microstructure and Mesoscopic Physics,*

Beijing Key Laboratory of Quantum Devices,

Peking University, Beijing 100871, China

[2]*Department of Physics, Southern University of Science*

and Technology of China, Shenzhen 518055, China

[3]*Department of Physics, Massachusetts Institute of Technology, Cambridge, MA 02139, USA*

[4]*Department of Physics, Ohio State University, Columbus, OH 43210, USA*

[5]*Department of Physics, University of California, Berkeley, CA 94720, USA*

[6]*Materials Sciences Division, Lawrence Berkeley National Laboratory, Berkeley, CA 94720, USA*

[7]*Condensed Matter Physics and Materials Science Department,*

Brookhaven National Laboratory, Upton, New York 11973, USA

[8]*Frontiers Science Center for Nano-optoelectronics and Collaborative Innovation Center,*

of Quantum Matter, Peking University, Beijing 100871, China

* These authors contributed equally.

† Corresponding Author. E-mail: liangfu@mit.edu (L.F.),

xswu@pku.edu.cn (X.W.),

zhangly@sustc.edu.cn (L.Z.)

SUPPLEMENTARY NOTE 1: EXPERIMENTAL DETAILS

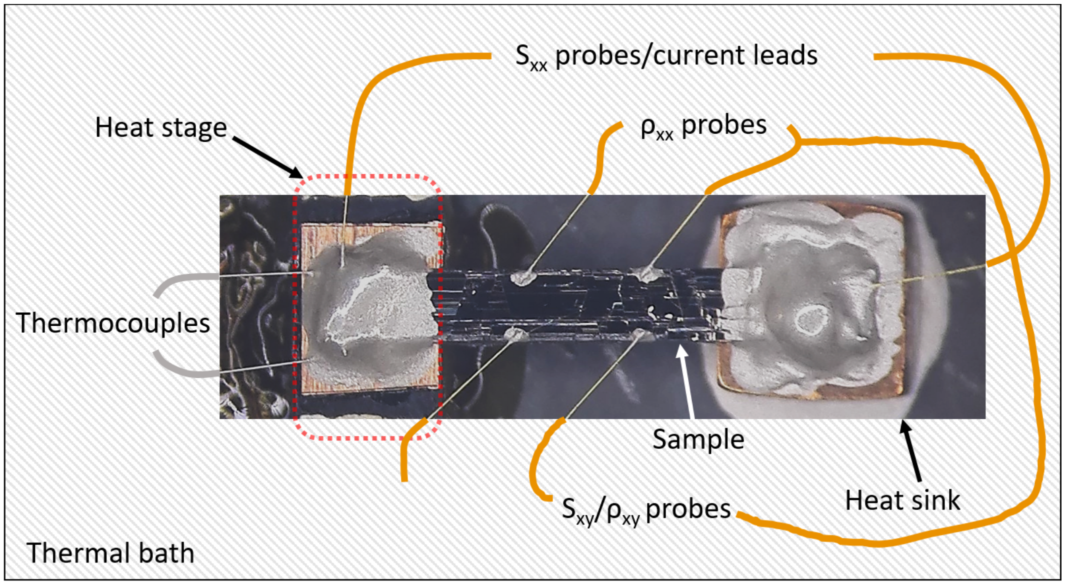

SUPPLEMENTARY FIG. 1: Optical image of our experimental setup. A chip heater (outlined by red dashed line) and a copper heat sink are mounted on the thermal bath by varnish and silver paste, respectively. A copper film is deposited on top of the heater to reduce temperature non-uniformity. Silver paste is used to make electrical contacts. 25 μm gold wires are used for electrical leads due to their small Seebeck coefficient. A Type-E thermocouple measures the temperature difference between the hot end of sample and the thermal bath, with one joint attaching to the sample and the other attaching to the pad on the thermal bath.

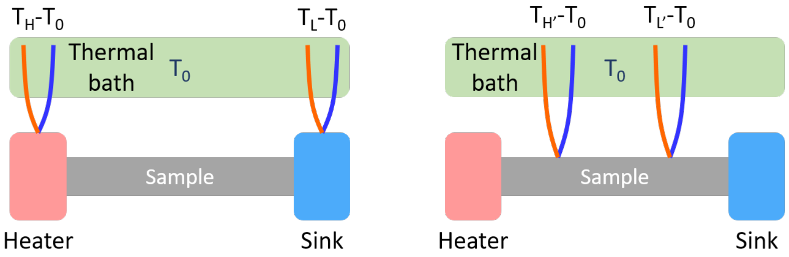

SUPPLEMENTARY FIG. 2: Schematics of two-point measurement (left) and four-point measurement (right) setups.

The optical image of our measurement setup is shown in Supplementary Fig. 1. We use a two-point method to measure the temperature difference between the hot end and cold end of

the sample (see Supplementary Fig. 2, left). Normally, two Type-E thermocouples are attached to the heater stage and the heat sink, respectively. The differential of the measured values between two stages is $\Delta T=(T_H-T_0)-(T_L-T_0)=T_H-T_L$. Here, T_H, T_L and T_0 are the temperature of the hot end, the heat sink and the thermal bath, respectively. In our experiment, only one thermocouple is used to get $T_H - T_0$, as the measured T_L-T_0 is found to be at most 3% of T_H-T_0 due to the good thermal contact between the copper sink and the thermal bath, meaning $\Delta T \approx (T_H-T_0)$. Thermoelectric voltages are measured through gold wires attached to two ends of the sample and the voltage probes in the middle. ρ_{xx} and ρ_{xy} are measured by a standard four-point method.

In general, there are two main types of thermoelectric measurement setups, i.e., two-point and four-point methods, shown in Supplementary Fig. 2. In a two-point setup, a sample is mounted such that it bridges a high temperature stage and a low temperature one. Thermometers are attached to the stages and measure the temperature difference ΔT between the two stages, while electric contacts attached to two ends of the sample are used to measure the thermoelectric voltage *V*. Since there is contact thermal resistance between the sample and the stages, and hence a temperature difference, the actual ΔT is smaller than the measured one, resulting in underestimation of the thermoelectric ratio *V*/ΔT. So, it is important to make good thermal contacts so that the contact thermal resistance is much smaller than the thermal resistance of the sample. A four-point method mitigates this problem by attaching thermometers directly to the sample. In such a case, the thermal contacts between the sample and the stages become irrelevant. However, this four-point method works only if the thermal resistance of the thermometer connection is much higher than both that of the sample and the contact thermal resistance between thermometers and the sample. If the former condition is not met, significant heat current will be drawn through the thermometers. This heat leakage gives rise to a non-uniform temperature gradient across the sample, affecting the calculation of the Nernst effect. If the latter condition is broken, the measured ΔT is smaller than the actual one, leading to an overestimation of the thermoelectric coefficients: the so-call cold finger effect.

$ZrTe_5$ is a van der Waals layered material. Moreover, in each layer, there are Zr-Te chains running along the *a* direction, while the interchain bonding is relatively weak, making it quasi-1D material. As a result, single crystals of $ZrTe_5$ are in the shape of a whisker or thin ribbon. Due to

the weak van der Waals interlayer coupling, crystals are soft and readily peeled off. These geometric and mechanical characteristics makes our thermoelectric measurements non-trivial. Furthermore, the thermal conductivity of ZrTe5 is relatively low, which, in combination with small cross-section of the sample, leads to a large thermal resistance. Consequently, it can be challenging to satisfy the condition for the four-point method. For this reason, a two-point method is preferable, especially when the sample is small [1]. As the sample is a ribbon, a large contact area between the sample and the stages, relative to the cross-section area of the sample, can be readily achieved. By further applying abundant silver paste, the contact thermal resistance can be made small. We have measured the temperature difference between one end of the sample and the thermal bath by attaching a thermocouple to that end. As shown in Supplementary Fig. 3, the difference is less than 10% of the measured ΔT above 40 K. This is not significant in a thermoelectric measurement. However, at low temperatures, it can amount to 18%. Consequently, the low temperature thermoelectric coefficients may be appreciably larger than the measured ones.

Let us now quantitatively estimate the heat leakage through the thermometers in a four-point method using the dimensions of our setup. The separation between the heater and heat sink is 1.35 mm. The cross-section of sample #1, presented in the main text, has an area 0.36 mm by 0.25 mm. The thermal conductivity of ZrTe5 is about 7-20 W/m·K in the temperature range of this study, according to previous reports [2]. The thermal conductivity reported by Constantan and Chromel, which, to the best of our knowledge, is only available at temperatures down to 100 K, are 19W/m·K and 13W/m·K at 100 K, respectively [3]. The thermocouples we used have a diameter of 25 μm with a length of about 3 mm limited by the chip carrier, on which the whole setup is mounted. Neglecting the unknown contact thermal resistance of the thermocouples and using the Fourier's law $JQ = -\kappa \nabla T$, the heat leakage through thermocouple wires is about 0.8 – 1.0% of the total heat through the sample at 100 -150 K. So, for this sample, the heat leakage is not a problem. However, for sample #2, presented in the Supplementary Information, the cross-section is only 0.25 mm by 0.04 mm, and the ratio reaches 7 - 9%.

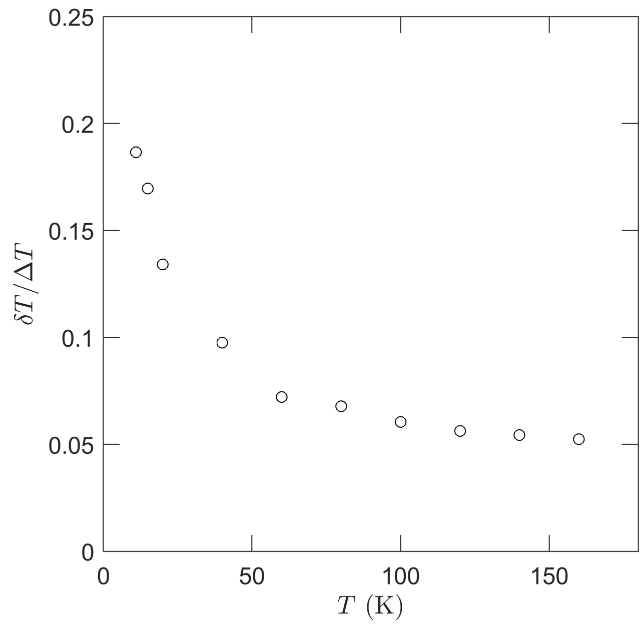

SUPPLEMENTARY FIG. 3: Estimation of the upper bound for the error of the measured temperature difference due to the contact thermal resistance between the cold end of the sample and the thermal bath. ΔT is the measured temperature difference between the hot end of the sample and the thermal bath, which is used to calculate the thermoelectric coefficients, while δT is the measured temperature difference between the cold end of the sample and the thermal bath. Above 50 K, the error is about 5%, but it increases to 18% at low temperature.

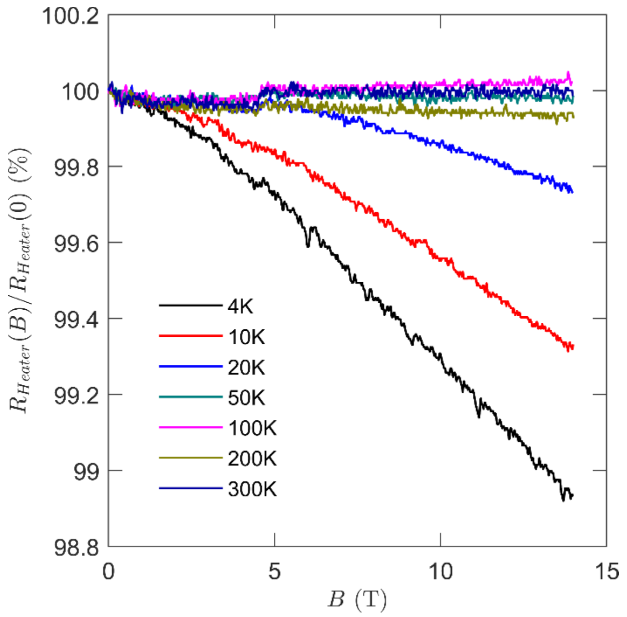

SUPPLEMENTARY FIG. 4: Magnetoresistance of the heater.

When applying a high magnetic field, the characteristics of thermometers may change. Care needs to be taken to obtain the correct ΔT. In this experiment, we used a Type-E thermocouple, which is field-independent above 20 K, although it gains some dependence below this temperature. To circumvent this issue, we adopted a strategy to make sure that ΔT is field independent so that there is no need to measure it in field. Since the heater resistance barely changes with field, as shown in Supplementary Fig. 4, the heating power remains unchanged under a constant current. Therefore, the key of the strategy is to make sure that the thermal resistance between two stages is much smaller than that of the sample. In this case ΔT does not vary with the latter. To achieve this requirement, the heater, consisting of a meandering thin metal strip on a 500 μm thick alumina substrate, is directly mounted on the thermal bath, using silver paste or varnish. On top of the heater, a thick copper film is deposited to ensure a uniform temperature on the stage. With this design, a heating power of 2.5– 8.1 mW was required to produce ΔT ~ 91 – 257 mK. The heat current through the sample is estimated as 12-53 μW, using the available thermal conductivity of $ZrTe_5$ [2] and the geometry of the sample. Apparently, only at most 1.5 % of the total heat current goes through the sample, which can have little effect on ΔT. The thermal conductance of alumina is unlikely to have any field dependence. In fact, above 20 K, where our thermocouple is field-independent, ΔT was found to be fairly constant in fields, as seen in Supplementary Fig. 5.

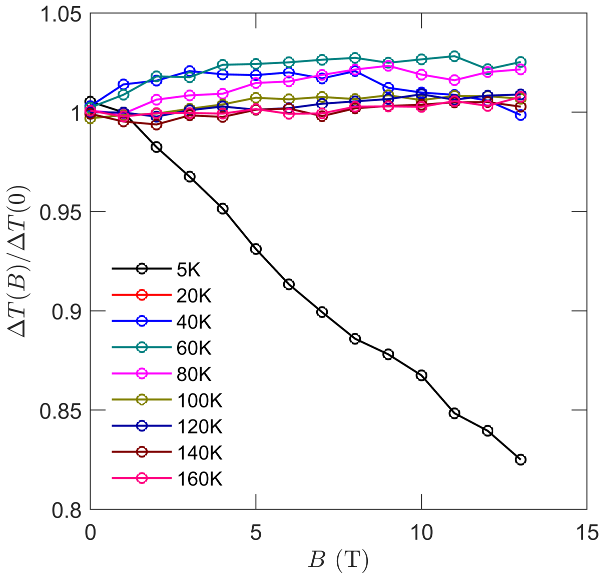

SUPPLEMENTARY FIG. 5: Field dependence of the thermoelectric signal of a thermocouple measuring the temperature difference between the heat stage and the thermal bath.

Under an applied magnetic field, the Nernst effect appears, which in this experiment is measured adiabatically. Under this condition, the Righi-Leduc effect generates a transverse temperature gradient, which in turn contributes a transverse electric field. This additional field has to be taken into account when obtaining the isothermal Nernst coefficient, which is used to calculate the thermoelectric Hall conductivity α_{xy} in this work. The Righi-Leduc effect is usually small. When the thermal conductivity of the material is dominated by the lattice contribution, the transverse temperature gradient is negligible. Then, the adiabatic Nernst coefficient is equivalent to the isothermal one [4]. This is in fact the case in $ZrTe_5$. The thermal conductivity of $ZrTe_5$ single crystals is about, 7 – 20 W/m·K according to previous reports [2]. Using the measured resistivity of our sample, we can estimate the electronic contribution to the thermal conductivity, based on the Wiedemann-Franz law, $\kappa/\sigma = LT$. It turns out to be ~ 0.05 – 0.7 W/m·K, only 3% of the total thermal conductivity. Therefore, our measurements directly produce the isothermal Nernst coefficient.

SUPPLEMENTARY NOTE 2: ANALYSIS OF MAGNETORESISTANCE

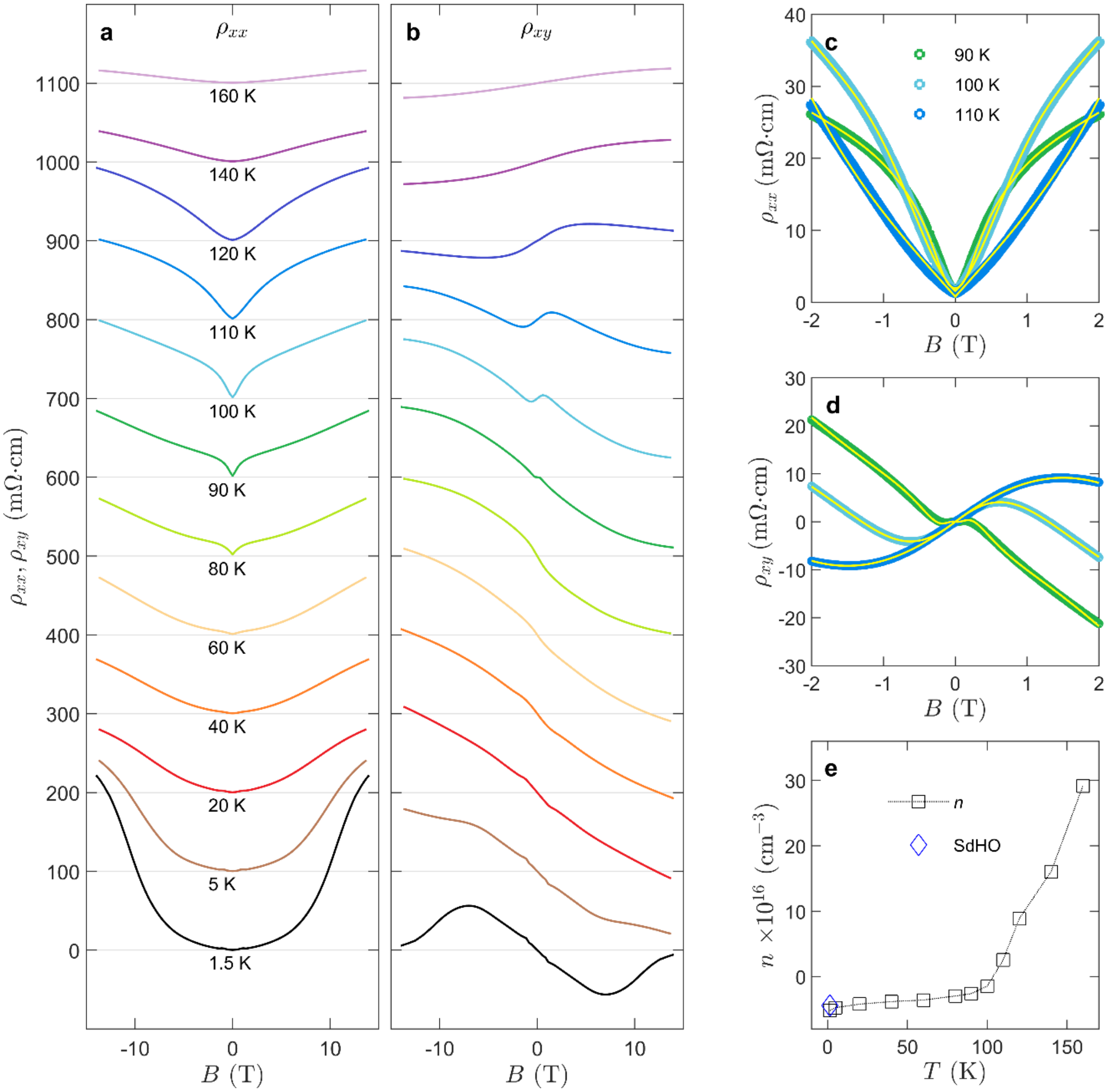

SUPPLEMENTARY FIG. 6: Electrical transport measurements. **a**, **b** Longitudinal electrical resistivity ρ_{xx} and transverse electrical (Hall) resistivity ρ_{xy} versus the magnetic field at different temperatures. Curves are shifted for clarity. **c**, **d** ρ_{xx} and ρ_{xy} at 90 K, 100 K, 110 K for $B < 2$ T. Bright solid lines are fits to a two band model. **e** Carrier densities as a function of temperature. Positive values represent holes, while negative values represent electrons. The diamond symbol represents the carrier density calculated from Shubnikov-de Haas oscillations at 1.5 K.

Supplementary Fig. 6 shows the magnetoresistance and the Hall resistance of the sample presented in the main text. The resistivity strongly increases with field, e.g., by two to three orders of magnitude at B = 14 T. Such a large magnetoresistance is typical for Dirac semimetals

[5]. At low temperatures, the Hall resistivity is rather linear below 6 T, indicating single band conduction. The linearity persists deep into the quantum limit. However, a strong deviation appears above 6 T. We believe that such a nonlinear Hall voltage in the extreme quantum limit is the result of two-carrier contributions, as expected when the Fermi level shifts towards the Dirac point with increasing magnetic field. This shifting of the Fermi level is precisely the mechanism that gives rise to the non-saturating thermopower and transverse thermoelectric conductivity plateau, which are the main focus of this work. The low-field slope of ρ_{xy} shows a sign-reversal at about 90 K, indicating the change of carrier from electron to hole, as expected by the Liftshitz transition in this material.

At temperatures that are much lower or much higher than 90 K, the Fermi level is not so close to the Dirac point and the transport is more or less one-carrier dominated at low fields. Correspondingly, the low-field Hall resistivity is relatively linear and the carrier density can be simply calculated from the Hall slope. On the other hand, around 90 K, where the Fermi level is in the vicinity of the Dirac point, a significant number of electrons and holes are thermally excited. The existence of two types of carriers with comparable densities causes ρ_{xy} to become non-linear even at relatively low fields. Consequently, a two-carrier model such that $\hat{\rho}_{tot}^{-1} = \hat{\rho}_{e}^{-1} + \hat{\rho}_{h}^{-1}$, is necessary to describe the magnetotransport and extract the carrier density. Here $\hat{\rho}_{\text{tot}}$, $\hat{\rho}_{\text{e}}$ and $\hat{\rho}_{\text{h}}$ are the resistivity tensors of the total, electron contribution and hole contribution, respectively. For the longitudinal component of the resistivity tensor, ρ_{xx}, we need to take into account the strong magnetoresistance that a Dirac semimetal often exhibits. The following generic expressions are used to describe both the electron and hole transport,

$$\begin{cases} \rho_{xx}(B) = \rho_0 + a\,|B| + bB^2 \\ \rho_{xy}(B) = R_{\mathrm{H}}B \end{cases} ,\ \hat{\rho} = \begin{pmatrix} \rho_{xx} & \rho_{xy} \\ -\rho_{xy} & \rho_{xx} \end{pmatrix} \tag{2-1}$$

By fitting data around 90 K, the carrier densities of holes and electrons, n_{h} and n_{e}, are obtained. Their difference, $n_{\text{h}} - n_{\text{e}}$, which reflects the Fermi level, is plotted in Supplementary Fig. 6e. The full results for n_h, n_e, μ_h, and μ_e are shown in Table 1. The temperature dependence of the carrier density is consistent with the Lifshitz transition observed in the angle resolve photo-emission spectroscopic study [6]. Overall, the mobility decreases with temperature, except for some deviation around 100 K. This is because a two-band model has to be used in this regime and increase in the number of fitting parameters leads to larger uncertainty.

Temperature (K)	1.5	5	20	40	60	80	90	100	110	120	140	160
n_h (10^{16} cm^{-3})	-	-	-	-	-	-	1.8	5.3	5.3	8.8	16.1	29.2
n_e (10^{16} cm^{-3})	5.2	4.7	4.2	3.8	3.6	3.0	4.4	6.8	2.8	-	-	-
μ_h (m^{2}V^{-1}s^{-1})	-	-	-	-	-	-	5.7	7.3	14.6	7.2	5.0	3.5
μ_e (m^{2}V^{-1}s^{-1})	42.9	64.0	55.3	35.8	16.9	10.2	4.9	0.1	0.2	-	-	-

SUPPLEMENTARY TABLE 1: Carrier density and mobility.

Supplementary Fig. 7 shows the oscillatory part of the magnetoresistance at 1.5 K, which is periodic in 1/*B*. At least six periods of oscillations can be identified. The FFT spectrum displays a sharp peak at about 0.82 T, which agrees well with the slope of the Landau plot shown in Fig. 1 of the main text. From the oscillation frequency, we estimate the carrier density 4.3×10^{16} cm^{-3}.

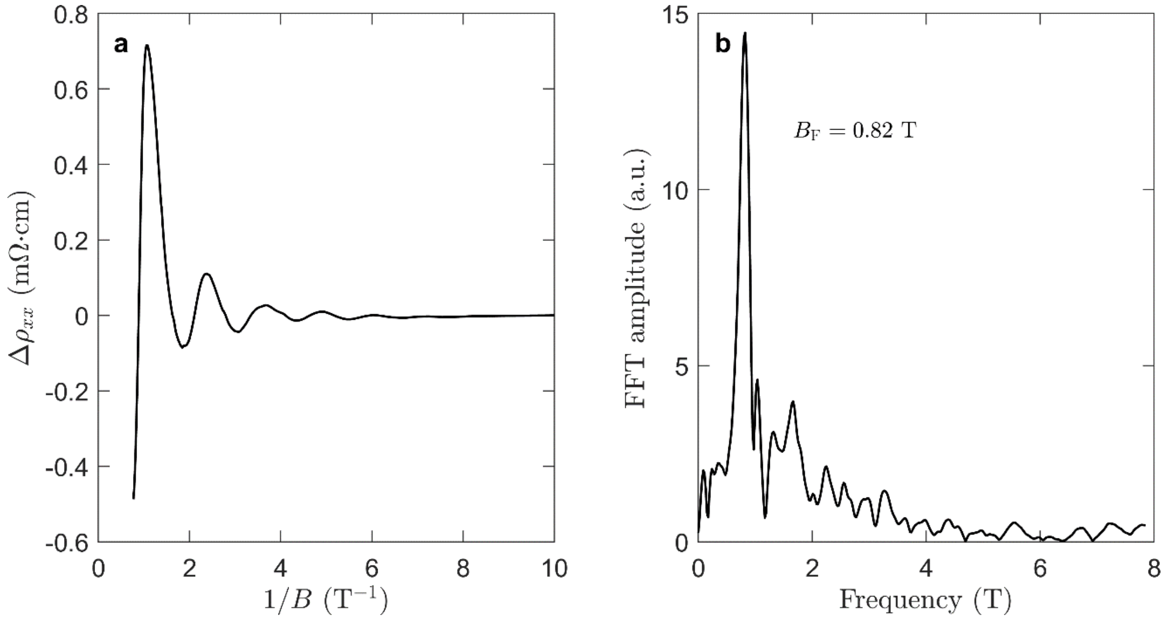

SUPPLEMENTARY FIG. 7: Quantum oscillations of magnetoresistance at 1.5 K. **a** Oscillations of the magnetoresistance after subtraction of a background. **b** Fast Fourier transformation analysis of the quantum oscillation.

SUPPLEMENTARY NOTE 3: ANALYSIS OF THERMOELECTRIC PROPERTIES

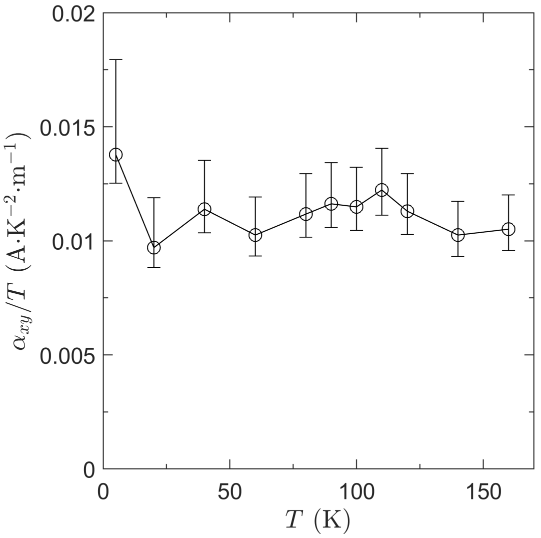

SUPPLEMENTARY FIG. 8: Thermoelectric conductivity α_{xy}/T at 14 T for different temperatures. The lowest temperature point shows a larger deviation. The error bars reflect uncertainties in measurements of the temperature difference and sample dimensions, and indicate one standard error. The uncertainty in the temperature difference is estimated from the plot in Supplementary Fig. 3.

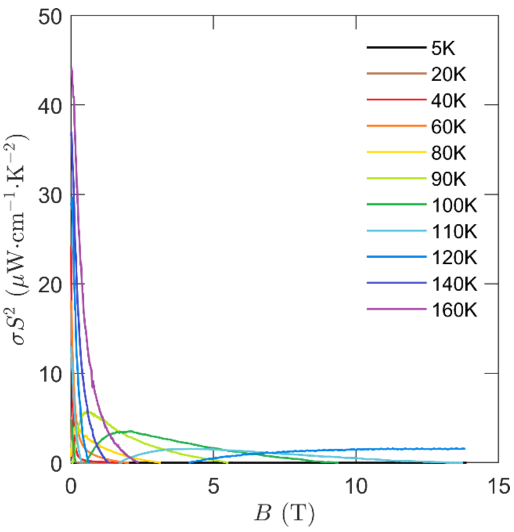

SUPPLEMENTARY FIG. 9: Power factor as a function of magnetic field. Overall, it decreases with increasing field. Due to the sign change of the Seebeck coefficient, the curves around 90 K are non-monotonic.

SUPPLEMENTARY NOTE 4: DATA FROM AN ADDITIONAL SAMPLE

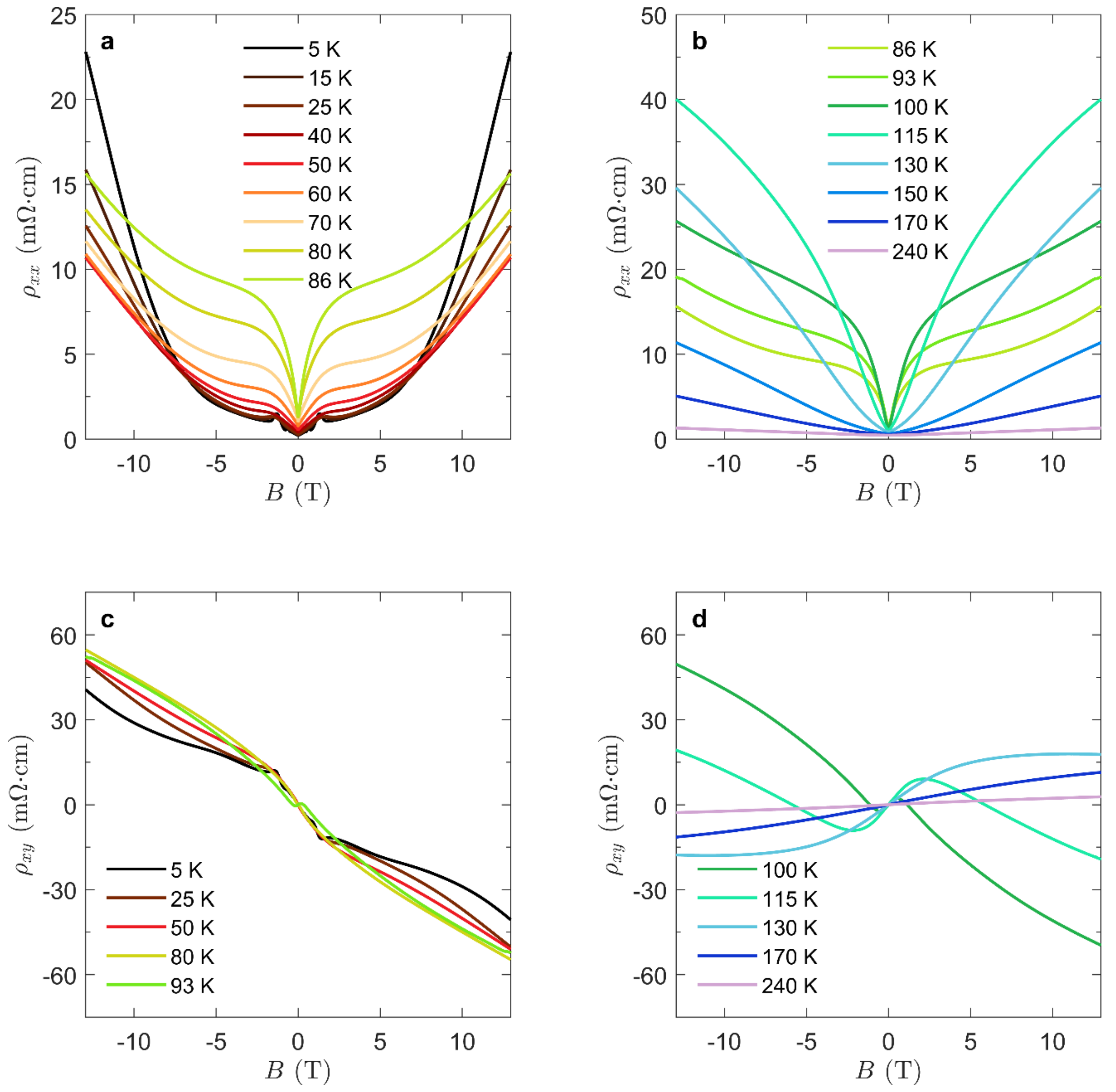

SUPPLEMENTARY FIG. 10: Electrical transport properties of sample #2. **a**, **b** Longitudinal electrical resistivity ρ_{xx} at different temperatures, showing strong magnetoresistance. At low temperatures, quantum oscillations appear at low fields. **c**, **d** Transverse electrical (Hall) resistivity ρ_{xy}. When the temperature is low, ρ_{xy} is linear in B, suggesting a single band. ρ_{xy} deviates from the linear dependence when the system is in the quantum limit. Around 100 K, the Hall resistivity becomes nonlinear, implying two-band transport.

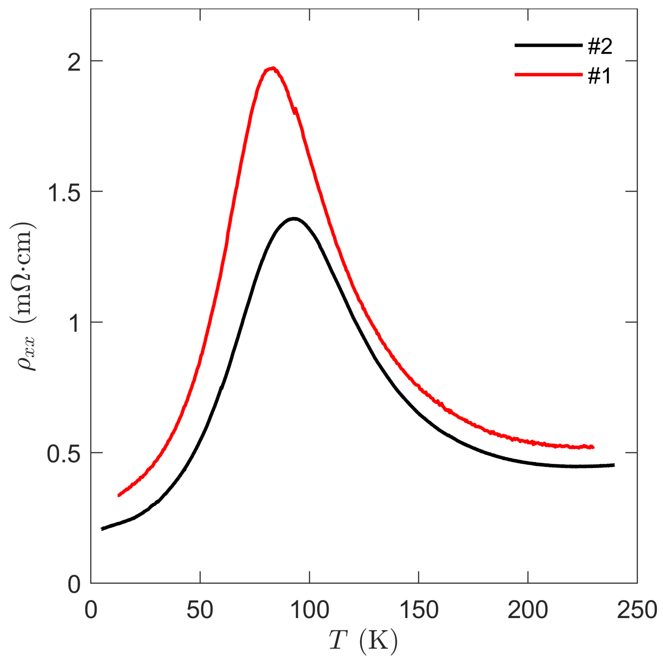

SUPPLEMENTARY FIG. 11: The zero-field resistivity ρ_{xx} as a function of temperature for sample #2 (lower, black curve) as compared to sample #1 (upper, red curve).

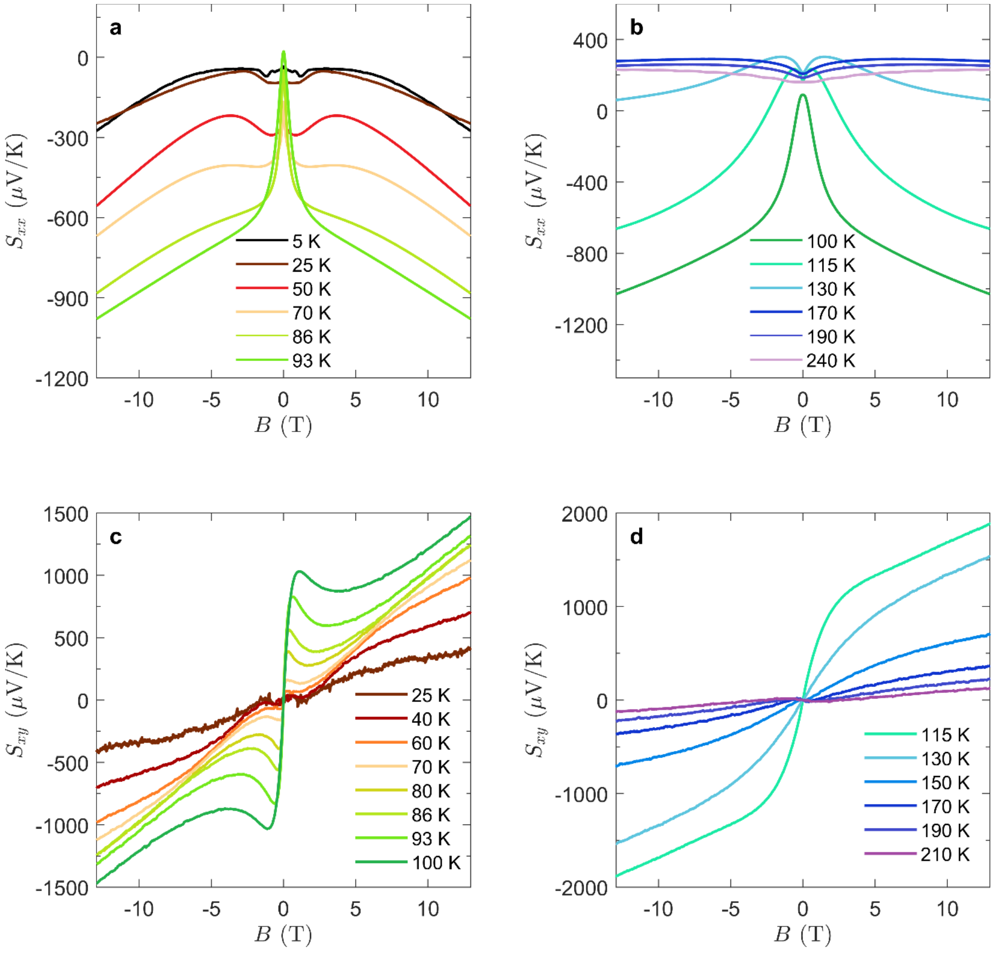

SUPPLEMENTARY FIG. 12: Thermoelectric coefficients of sample #2. **a**, **b** S_{xx} at different temperatures. **c**, **d** S_{xy}.

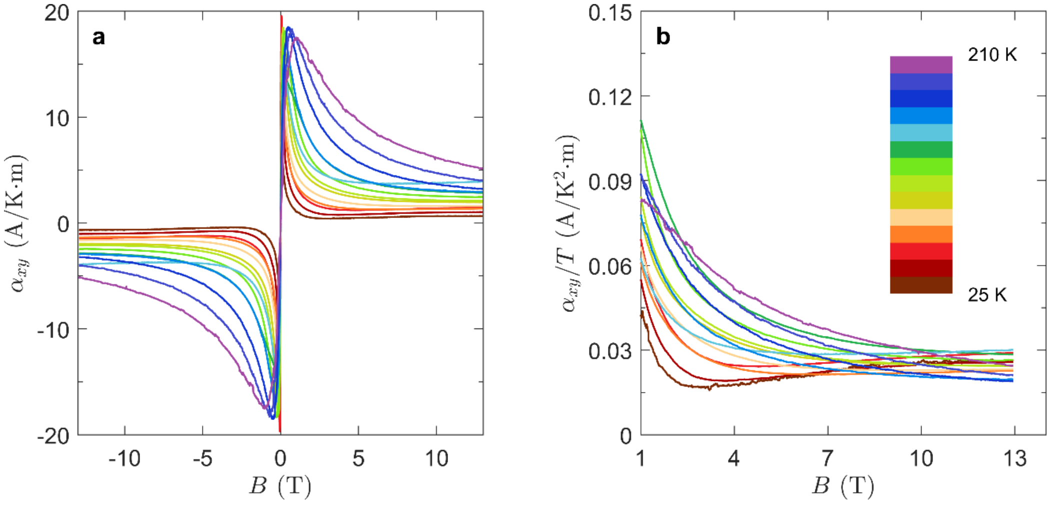

SUPPLEMENTARY FIG. 13 Transverse thermoelectric conductivity of sample #2. **a**, α_{xy} as a function of B at different temperatures. **b**, Plateau of α_{xy}/T at high magnetic field.

Supplementary Figs. 10-13 show data taken from another sample, which we dub sample #2. Overall, the results are very similar to the sample ("sample #1") presented in the main text. The temperature dependence of the longitudinal electrical resistivity has a maximum at about 93 K. At 1.5 K, the sample mobility is about 370,000 $cm^2V^{-1}s^{-1}$. In the field dependence of the resistivity, quantum oscillations are well developed at low magnetic fields. The quantum limit is reached above 2 T, slightly higher than for the sample in the main text, suggesting a slightly larger value of the electron concentration at B=0. ρ_{xy} is linearly dependent on B below the quantum limit at low temperatures, while it becomes nonlinear around the temperature of the resistivity maximum. These features are consistent with the Liftshiz transition discussed in the main text. One can notice that the magnetoresistance effect is somewhat weaker in sample #2 as compared to sample #1, particularly at high fields ~ 10 T. In our samples, one of the primary drivers of magnetoresistance at such high fields is the variation in carrier concentration with field arising from the Lifshitz transition. In sample #2 achieving a large variation seems to require a relatively higher value of magnetic field as compared to sample #1, which is consistent with the sample #2's larger electron concentration at B = 0.

Supplementary Fig. 12 shows the thermoelectric coefficients, S_{xx} and S_{xy}. The results in the main text are reproduced in this sample. Both coefficients exhibit a mostly linear increase in the quantum limit, with S_{xy} becoming as large as 1900 μV/K. Supplementary Fig. 13 shows the transverse thermoelectric conductivity. The value of α_{xy}/T at different temperatures converges to a plateau value in the range 0.02 – 0.03 A/K^2·m at high magnetic field. Although this value is higher than the one in the main text, it is still in reasonably good agreement with the theory. The difference between samples may be explained by variation of the Fermi velocity among samples due to high sensitivity of the band structure on the lattice constant, or by slight differences in alignment with the magnetic field (note that $ZrTe_5$ has a strongly anisotropic Fermi velocity). We reiterate that the variation of the band structure of $ZrTe_5$ is manifested in many experiments with various techniques [7], with three different topological phases being reported. Thinning the thickness of a crystal down to 180 nm can increase the band gap from zero to 10 meV, even though no quantum confinement is expected at this thickness [8]. The peak in the temperature dependence of the resistivity, one of the characteristic features of $ZrTe_5$, can vary from 0 to 140 K. Therefore, it is not surprising that the Fermi velocity may vary from sample to sample. Additionally, the apparent deviation from the universal plateau value at low

temperatures may be related to the presence of a small band gap, as we discuss theoretically in Sec. VI.

SUPPLEMENTARY NOTE 5: DISCUSSION OF POSSIBLE PHONON DRAG EFFECTS

The expression for α_{xy} presented in the main text, Eq. (1), is derived under the assumption that there is no significant phonon contribution to the thermoelectric conductivity. Here we discuss the possible role of phonons. In particular, we discuss whether phonon drag effects may be responsible for the slight upturn in α_{xy} that is suggested by Fig. 3a and Supplementary Fig. 11b with increasing field at the lowest temperatures.

While phonon modes are typically electroneutral, and have no direct coupling to magnetic field, they may still contribute to α_{xy} through the phonon drag effect: when an electric current is present, electron-phonon scattering processes give the phonon system a net drift velocity. Thus, an electron heat current may be accompanied by a collinear phonon heat current. Such a phonon current would tend to increase the thermoelectric conductivity.

One can make an upper-bound estimate for the effect of phonon drag by assuming that the phonon drift velocity is identical to the electron drift velocity. This strongest-case scenario corresponds to the limit of strong electron-phonon scattering and weak phonon-phonon or phonon-impurity scattering. [9] As explained in the main text, the heat current at large magnetic field is directly proportional to the entropy density. Thus, in this same limit the contribution of phonon drag effects to α_{xy} is directly proportional to the phonon entropy. In the limit of low temperature the phonon entropy is dominated by acoustic phonons, which have an entropy

$$S_{ph} \sim \frac{k_B^4 T^3}{\hbar^3 v_s^3} \sim \frac{k_B}{V_0}\left(\frac{T}{\Theta_D}\right)^3 \tag{5-1}$$

per unit volume, where v_s is the speed of sound, V_0 is the unit cell volume, and Θ_D is the Debye temperature. As discussed in the main text, the electron entropy in the EQL is of order

$$S_{el} \sim \frac{N_f k_B^2 T e B}{\hbar^3 v_F}. \tag{5-2}$$

Thus, at low temperatures and in the EQL the relative contribution of phonon drag to α_{xy} can be no larger than

$$\left(\frac{\alpha_{xy}^{\text{(phonon drag)}}}{\alpha_{xy}^{\text{(electrons)}}}\right) < \frac{S_{ph}}{S_{el}} \sim \frac{\hbar^2 v_F}{N_f k_B T V_0 e B}\left(\frac{T}{\Theta_D}\right)^3. \tag{5-3}$$

For $ZrTe_5$ the Debye temperature $\Theta_D \approx 150$ K [10] and the unit cell volume $V_0 \approx 400$ Å^3.

While this estimate for $\alpha_{xy}^{(\text{phonon drag})}/\alpha_{xy}^{(\text{electrons})}$ gives a numerical value as large as several tenths within the EQL, it predicts a temperature and field dependence that is inconsistent with our observations. In particular, Supplementary Eq. (5-3) suggests that the contribution of phonon drag is larger at higher temperatures and at lower fields. On the other hand, the slight deviation from the plateau value of α_{xy} becomes more pronounced in the opposite limit of lower temperatures and at higher fields. Thus, we find it more likely that the slight deviation in α_{xy} from the plateau value is associated with the presence of a very small gap. We discuss this possibility and its consequences in the following section.

SUPPLEMENTARY NOTE 6: THEORETICAL CALCULATION OF α_{xy} FOR THE CASE OF A MASSIVE DIRAC DISPERSION

In this section we present a theoretical calculation of the thermoelectric Hall conductivity α_{xy} for the case of a massive Dirac dispersion. This analysis is a straightforward generalization of the results obtained in Ref. [11] to the case of a finite band gap.

The energy of a massive Dirac particle in a magnetic field is given by the expression

$$\varepsilon_n(k_z) = \mathrm{sign}(n) \cdot \sqrt{\Delta^2 + 2e\hbar B|n|v_\perp^2 + v_F^2\hbar^2k_z^2} \tag{6-1}$$

where 2Δ is the band gap, v_F is the Fermi velocity in the magnetic field direction z, and $v_\perp$ is the velocity in plane perpendicular to the field direction (more carefully, it is the geometric mean of v_x and v_y). The density of states is then given by

$$\nu(\varepsilon) = \frac{N_f Be}{\pi^2\hbar^2 v_F}\mathrm{Re}\left(\frac{|\varepsilon|}{2\sqrt{\varepsilon^2-\Delta^2}} + \sum_{n=1}^{\infty}\frac{|\varepsilon|}{\sqrt{\varepsilon^2-\Delta^2-2\hbar v_\perp^2 eBn}}\right) \tag{6-2}$$

where N_f is the number of Dirac nodes (each of the nodes is double degenerate).

The thermoelectric Hall conductivity is determined by the expression [11]

$$\alpha_{xy} = \frac{eN_f}{\pi\hbar}{\sum_{n=0}^{\infty}}' \int_0^\infty \frac{dk_z}{\pi}\left[s\left(\frac{\varepsilon_n(k_z)-\mu}{k_BT}\right) + s\left(\frac{\varepsilon_n(k_z)+\mu}{k_BT}\right)\right] \tag{6-3}$$

where μ is the chemical potential, and T is the temperature. The notation ${\sum_{n=0}^{\infty}}'$ is used to mean that there is an extra factor 1/2 multiplying the n = 0 term of the sum, and $\varepsilon_0(k_z)$ should be understood as $\varepsilon_0(k_z) = \sqrt{\Delta^2 + v_F^2\hbar^2k_z^2}$ in the above expression. The entropy per electron state is given by

$$s(x) = -k_B\left[n_F \ln n_F + (1-n_F)\ln(1-n_F)\right] = k_B\left[\ln\left(1+e^x\right) - \frac{x}{1+e^{-x}}\right] \tag{6-4}$$

where $n_F(\varepsilon) = [1 + \exp(\varepsilon/k_BT)]^{-1}$ is the Fermi-Dirac distribution.

Finally, the chemical potential μ should be determined self-consistently from the equation

$$\int_0^{\infty} d\varepsilon\, \nu(\varepsilon) n_F(\varepsilon - \mu) - \int_{-\infty}^{0} d\varepsilon\, \nu(\varepsilon) \left[1 - n_F(\varepsilon - \mu)\right] = n_0 \tag{6-5}$$

where n_0 is net electron concentration.

The equations in this section fully determine the thermoelectric Hall conductivity α_{xy} as a function of magnetic field B, temperature T, and electron concentration n_0. An example of the solution is shown in Supplementary Fig. 14. Note, in particular, that at temperatures much smaller than the band gap and at sufficiently high magnetic fields, the electron dispersion near the Fermi level is essentially that of Schrodinger particles, which do not exhibit a quantized plateau [11].

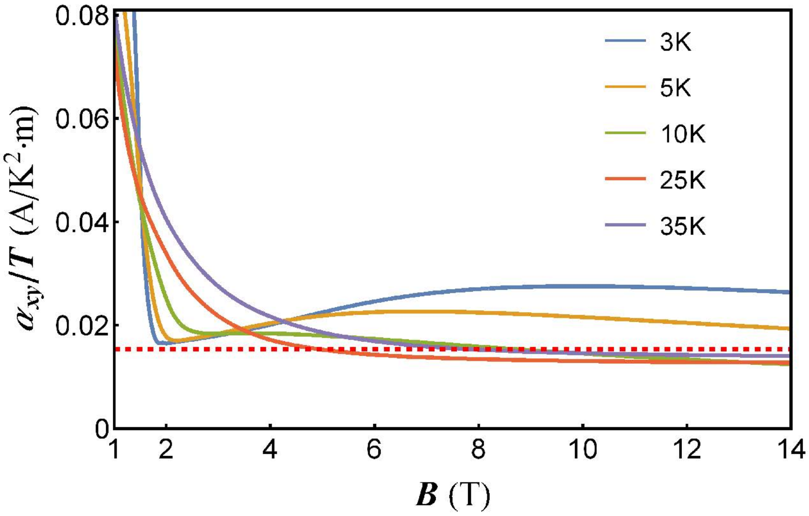

SUPPLEMENTARY FIG. 14: An example calculation of α_{xy} as a function of B for different temperatures T. This result is obtained as a solution of Supplementary Eqs. (6-1) through (6-5) with $n_0 = 5 \times 10^{16}$ cm^{-3}, $N_f = 1$, $v_F = 3 \times 10^4$ m/s, $v_\perp = 6v_F$, and $\Delta/k_B = 30$K. The red dashed line indicates the position of the plateau for the same parameter values when $\Delta = 0$, i.e., $\alpha_{xy}^{\text{plateau}} = Tek_B^2 N_f/6v_F\hbar^2$.

SUPPLEMENTARY REFERENCES

[1] Bartosz M. Zawilski, Roy T. Littleton IV, and Terry M. Tritt, Rev. Sci. Instrum. **72**, 1770 (2001).

[2] B. M. Zawilski, R. T. Littleton IV, and Terry M. Tritt, Appl. Phys. Lett. 77, 2319 (2000).

[3] B. Sundqvist, J. Appl. Phys. 72.2, 539 (1992).

[4] K. Sugihara, J. Phys. Soc. Jpn. 27, 362 (1969).

[5] T. Liang, Q. Gibson, M. N. Ali, M. Liu, R. J. Cava, and N. P. Ong, Nature Materials **14**, 280 (2014).

[6] Y. Zhang, C. Wang, L. Yu, G. Liu, A. Liang, J. Huang, S. Nie, X. Sun, Y. Zhang, B. Shen, J. Liu, H. Weng, L. Zhao, G. Chen, X. Jia, C. Hu, Y. Ding, W. Zhao, Q. Gao, C. Li, S. He, L. Zhao, F. Zhang, S. Zhang, F. Yang, Z. Wang, Q. Peng, X. Dai, Z. Fang, Z. Xu, C. Chen, and X. J. Zhou, Nature Communications **8**, 15512 (2017).

[7] P. Shahi, D. J. Singh, J. P. Sun, L. X. Zhao, G. F. Chen, Y. Y. Lu, J. Li, J.-Q. Yan, D. G. Mandrus, and J.-G. Cheng, Phys. Rev. X **8**, 021055 (2018).

[8] Zhi-Guo Chen, R. Y. Chen, R. D. Zhong, John Schneeloch, C. Zhang, Y. Huang, Fanming Qu, Rui Yu, Q. Li, G. D. Gu, and N. L. Wang, PNAS **114**, 816 (2017).

[9] J. M. Ziman, *Principles of the Theory of Solids*, Cambridge University Press, 1972.

[10] Jie Zhu, Tianli Feng, Scott Mills, Peipei Wang, Xuewang Wu, Liyuan Zhang, Sokrates T. Pantelides, Xu Du, Xiaojia Wang, ACS Appl. Mater. Interfaces **10**, 40740 (2018).

[11] V. Kozii, B. Skinner, and L. Fu, Phys. Rev. B **99**, 155123 (2019).
